# Gatifloxacin versus chloramphenicol for uncomplicated enteric fever: an open-label, randomised, controlled trial

**DOI:** 10.1016/S1473-3099(11)70089-5

**Published:** 2011-06

**Authors:** Amit Arjyal, Buddha Basnyat, Samir Koirala, Abhilasha Karkey, Sabina Dongol, Krishna Kumar Agrawaal, Nikki Shakya, Kabina Shrestha, Manish Sharma, Sanju Lama, Kasturi Shrestha, Nely Shrestha Khatri, Umesh Shrestha, James I Campbell, Stephen Baker, Jeremy Farrar, Marcel Wolbers, Christiane Dolecek

**Affiliations:** a Oxford University Clinical Research Unit–Patan Academy of Health Sciences, Kathmandu, Nepal; b The Hospital for Tropical Diseases, Wellcome Trust Major Overseas Programme, Oxford University Clinical Research Unit, Ho Chi Minh City, Vietnam; c Centre for Tropical Medicine, University of Oxford, Oxford, UK; d London School of Hygiene and Tropical Medicine, London, UK

## Abstract

**Background:**

We aimed to investigate whether gatifloxacin, a new generation and affordable fluoroquinolone, is better than chloramphenicol for the treatment of uncomplicated enteric fever in children and adults.

**Methods:**

We did an open-label randomised superiority trial at Patan Hospital, Kathmandu, Nepal, to investigate whether gatifloxacin is more effective than chloramphenicol for treating uncomplicated enteric fever. Children and adults clinically diagnosed with enteric fever received either gatifloxacin (10 mg/kg) once a day for 7 days, or chloramphenicol (75 mg/kg per day) in four divided doses for 14 days. Patients were randomly allocated treatment (1:1) in blocks of 50, without stratification. Allocations were placed in sealed envelopes opened by the study physician once a patient was enrolled into the trial. Masking was not possible because of the different formulations and ways of giving the two drugs. The primary outcome measure was treatment failure, which consisted of at least one of the following: persistent fever at day 10, need for rescue treatment, microbiological failure, relapse until day 31, and enteric-fever-related complications. The primary outcome was assessed in all patients randomly allocated treatment and reported separately for culture-positive patients and for all patients. Secondary outcome measures were fever clearance time, late relapse, and faecal carriage. The trial is registered on controlled-trials.com, number ISRCTN 53258327.

**Findings:**

844 patients with a median age of 16 (IQR 9–22) years were enrolled in the trial and randomly allocated a treatment. 352 patients had blood-culture-confirmed enteric fever: 175 were treated with chloramphenicol and 177 with gatifloxacin. 14 patients had treatment failure in the chloramphenicol group, compared with 12 in the gatifloxacin group (hazard ratio [HR] of time to failure 0·86, 95% CI 0·40–1·86, p=0·70). The median time to fever clearance was 3·95 days (95% CI 3·68–4·68) in the chloramphenicol group and 3·90 days (3·58–4·27) in the gatifloxacin group (HR 1·06, 0·86–1·32, p=0·59). At 1 month only, three of 148 patients were stool-culture positive in the chloramphenicol group and none in the gatifloxacin group. At the end of 3 months only one person had a positive stool culture in the chloramphenicol group. There were no other positive stool cultures even at the end of 6 months. Late relapses were noted in three of 175 patients in the culture-confirmed chloramphenicol group and two of 177 in the gatifloxacin group. There were no culture-positive relapses after day 62. 99 patients (24%) experienced 168 adverse events in the chloramphenicol group and 59 (14%) experienced 73 events in the gatifloxacin group.

**Interpretation:**

Although no more efficacious than chloramphenicol, gatifloxacin should be the preferred treatment for enteric fever in developing countries because of its shorter treatment duration and fewer adverse events.

**Funding:**

Wellcome Trust.

## Introduction

Enteric fever is a disease that predominantly affects children and is caused by the faecal–oral transmission^1^ of *Salmonella enterica* serotype Typhi (*S typhi*) and *Salmonella enterica* Paratyphi A (*S paratyphi* A). There are an estimated 26 million infections and over 200 000 deaths caused by the disease worldwide each year.^2^ In parts of south Asia, the incidence of enteric fever in children can be as high as 573 cases per 100 000 person years.^3^

Chloramphenicol was the standard treatment for enteric fever from the 1950s^1^, ^4^, ^5^ until the development and spread of multidrug resistant (MDR; defined as resistance to all first-line antibiotics: chloramphenicol, amoxicillin, and co-trimoxazole) *S typhi* and *S paratyphi* A in the early 1990s. Subsequently, fluoroquinolones became first choice for the treatment of enteric fever. However, increased resistance to the older generation fluoroquinolones (ciprofloxacin and ofloxacin) has emerged. This reduces the options for treatment, and raises the spectre of fully resistant enteric fever.^1^, ^6^

Conflicting reports have emerged from randomised controlled trials with relatively small sample sizes that assessed older fluoroquinolones (ciprofloxacin and ofloxacin) versus chloramphenicol for the treatment of enteric fever.^1^, ^7^ Additionally, no trials have been done to investigate the efficacy of chloramphenicol versus a newer fluoroquinolone, such as gatifloxacin, in the treatment of enteric fever in children.^1^, ^8^ Recent reports suggest a general decline in the prevalence of MDR typhoid fever in Asia,^9^, ^10^, ^11^, ^12^, ^13^, ^14^, ^15^ and two recent studies of patients with enteric fever in Kathmandu, Nepal reported a low prevalence of chloramphenicol resistance in *S typhi* and *S paratyphi* A isolates: nine (1·7%) in 522 strains of *S typhi*^16^ and three (1·2%) of 247 strains of *S paratyphi* A.^10^

Gatifloxacin was effective in the treatment of nalidixic-acid-resistant enteric fever in two previous randomised trials done in Nepal^16^ and Vietnam.^17^ The drug targets both DNA gyrase and topoisomerase IV,^18^, ^19^ and hence is less inhibited by the common mutations of the *gyrA* gene of *S typhi* than are ciprofloxacin or ofloxacin.

We designed a randomised controlled trial to assess whether gatifloxacin had superior efficacy compared with chloramphenicol in adults and children with uncomplicated enteric fever in Nepal.

## Methods

### Patients

The study physicians enrolled patients who presented to the outpatient or emergency department of Patan Hospital, Lalitpur, Nepal from May 2, 2006, to August 30, 2008. Patients with fever for more than 3 days who were clinically diagnosed to have enteric fever (undifferentiated fever with no clear focus of infection on preliminary physical exam and laboratory tests) whose residence was in a predesignated area of about 20 km^2^ in urban Lalitpur and who gave fully informed written consent were eligible for the study. Exclusion criteria were pregnancy or lactation, age under 2 years or weight less than 10 kg, shock, jaundice, gastrointestinal bleeding, or any other signs of severe typhoid fever, previous history of hypersensitivity to either of the trial drugs, or known previous treatment with chloramphenicol, quinolone antibiotic, third generation cephalosporin, or macrolide within 1 week of hospital admission. Patients who had received amoxicillin or co-trimoxazole were included as long as they did not show evidence of clinical response. Ethical approval was granted by both Nepal Health Research Council and Oxford Tropical Research Ethics Committee.

### Randomisation and masking

Randomisation was done in blocks of 50 without stratification by an administrator otherwise not involved in the trial. The random allocations were placed in sealed opaque envelopes, which were kept in a locked drawer and opened by the study physician once each patient was enrolled into the trial after meeting the inclusion and exclusion criteria. Patients were enrolled in the order they presented and the sealed envelopes were opened in strict numerical sequence. Masking was not possible because of the different formulations and ways of giving the two drugs.

### Procedures

Each enrolled patient was randomly assigned to treatment with either gatifloxacin tablets (400 mg) 10 mg per kg per day in a single oral dose for 7 days or chloramphenicol capsules (250 mg or 500 mg) 75 mg per kg per day in four divided oral doses for 14 days. Gatifloxacin tablets were cut and weighed and the patients' daily doses were prepared in sealed plastic bags. The per-protocol planned duration of chloramphenicol treatment of 14 days was modified for blood-culture-negative patients, who received at least 8 days of chloramphenicol and stopped either on day 8 or 5 days after being afebrile, whichever came later. Gatifloxacin was given for 7 days in all patients.

After enrolment, patients were managed as outpatients and seen by trained community medical auxiliaries (CMAs), as described previously.^16^ The CMAs made a visit to each patient's house every 12 h for either 10 days (gatifloxacin group), 14 days (chloramphenicol group), or until the patient was cured. The CMA directly observed each patient ingesting the single dose of gatifloxacin and two doses of chloramphenicol. The physicians re-examined the patients on days 8 and 15, and at 1, 3, and 6 months. All examinations were standardised and entered into case record forms.

Complete blood counts were done on days 1, 8, and 15. On day 1, serum creatinine, bilirubin, aspartate aminotransferase (AST), and alanine aminotransferase (ALT) were also checked. Random plasma glucose was measured on day 1, day 8, day 15, and 1 month. On days 2–7, during the evening visit, the blood glucose was measured by finger-prick testing (OneTouch SureStep, Johnson and Johnson, USA) by the CMAs. Haemoglobin A_1C_ was measured at 3 months.

Blood culture was done as described previously^16^ in all patients at admission, in the culture-positive patients on day 8, and if symptoms and signs suggested further infection.

Stool cultures were done on admission in all patients, and in culture-positive patients after completion of treatment and at the 1 month, 3 month, and 6 month visits in 10 mL of Selenite F broth and incubated at 37°C. After the overnight incubation, the broth was subcultured onto MacConkey agar and xylose lysine decarboxylase agar media.

Isolates were screened using standard biochemical tests, and *S typhi* and *S paratyphi* A were identified using API20E (BioMerieux, Paris, France) and slide agglutination with specific antisera (MurexBiotech, Dartford, UK).

Minimum inhibitory concentrations (MICs) were calculated for amoxicillin, azithromycin, chloramphenicol, co-trimoxazole, nalidixic acid, ofloxacin, ciprofloxacin, tetracycline, gatifloxacin, and ceftriaxone by E-test (AB Biodisk, Solna, Sweden).

The primary endpoint of this study was the composite endpoint of treatment failure, which consisted of any one of the following: persistence of fever of more than 37·5°C at day 10 of treatment; the need for rescue treatment with ceftriaxone or ofloxacin as judged by the treating physician; microbiological failure, defined as a positive blood culture for *S typhi* or *S paratyphi* A on day 8; relapse, that is reappearance of culture-confirmed (including mismatch of serotypes [eg, day 1 blood culture positive for *S typhi* and relapse blood culture positive for *S paratyphi* A or vice versa]) or syndromic enteric fever on or after day 11 to day 31 in patients who were initially categorised as successfully treated; and occurrence of enteric-fever-related complications.^16^ Time to treatment failure was defined as the time from the first dose of treatment until the date of the earliest failure event of that patient, and patients without an event were censored at the date of their last follow-up visit.

Secondary endpoints were fever clearance time (FCT: time from the first dose of treatment given until the temperature was ≤37·5°C and the patient remained afebrile for at least 48 h); time to relapse until day 31, day 62, or month 6 of follow-up; and faecal carriage at the follow-up visits at 1, 3, and 6 months. The patients' FCTs were calculated electronically on the basis of twice-daily recorded temperatures. Patients without recorded fever clearance or relapse were censored at the date of their last follow-up visit. To reduce possible bias, an investigator not involved in the recruitment of patients decided patients' final outcomes by use of a masked database.

### Statistical analysis

The trial was designed as a superiority trial with the hypothesis that gatifloxacin was superior to chloramphenicol in patients with enteric fever. The sample size was calculated to detect a difference of 10% between the two groups in the proportion of patients reaching treatment failure at the two-sided 5% significance level with 80% power. We assumed treatment failure rates of 15% in the chloramphenicol and 5% in the gatifloxacin group, leading to a total required sample size of 160 patients with culture-confirmed enteric fever per group—320 patients in total. On the basis of results from a previous study,^10^, ^16^ we assumed that about 40% of patients who were randomly assigned treatment had culture-confirmed enteric fever. To allow for a loss to follow-up rate of about 5%, a total of 853 patients with suspected enteric fever were recruited to the trial.

Times to treatment failure, fever clearance, and relapse, were analysed by use of survival methods. The cumulative incidence of events was calculated with the Kaplan-Meier method, and comparisons were based on Cox regression models with the treatment group as the only covariate. For the primary endpoint (treatment failure), we also compared the absolute risk of treatment failure until day 31 on the basis of Kaplan-Meier estimates and standard errors according to Greenwood's formula.^20^ Additionally, the time to treatment failure was analysed in the subgroups defined by culture result, pathogen (*S typhi* or *S paratyphi* A), and age (<16 years or ≥16 years), and heterogeneity of the treatment effect was tested with a Cox regression model that included an interaction between treatment and subgroup.

The per-protocol analysis population consisted of all patients with blood-culture-confirmed enteric fever. We also analysed all patients who were assigned treatment, with the exception of those patients who were mistakenly randomised or withdrew before the first dose of study treatment, for treatment failure and safety.

All reported tests were done at the two-sided 5% significance level, and 95% CIs are reported. All analyses were done with the statistical software R version 2.9.1.^21^

The trial is registered on controlled-trials.com, number ISRCTN 53258327.

### Role of the funding source

The sponsor of the study had no role in study design, data collection, data analysis, data interpretation, or writing of the report. The corresponding author had full access to all the data in the study and had final responsibility for the decision to submit for publication.

## Results

**Figure 1:**
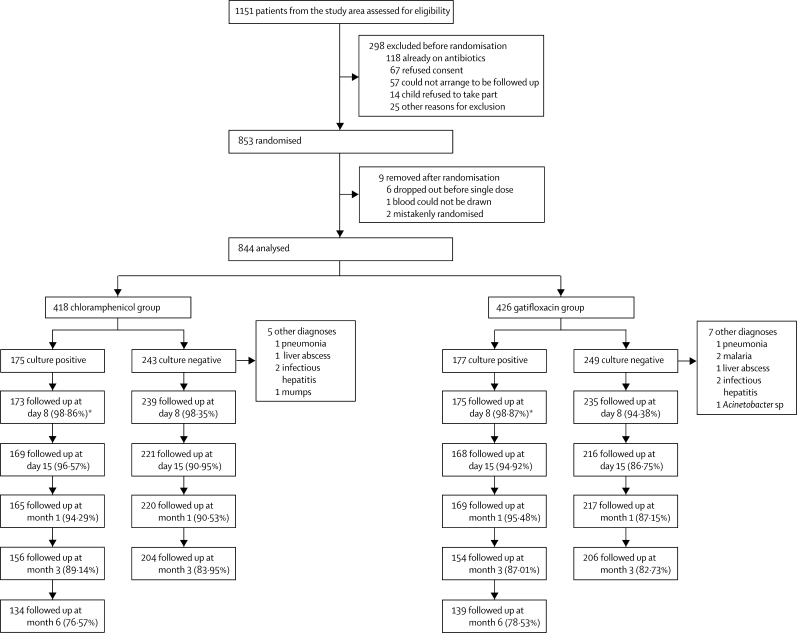
Trial profile *Two culture-positive patients in both the chloramphenicol and glatifloxacin groups were lost to follow-up before day 8.

Of 1151 patients assessed, 853 were assigned treatment; 844 were analysed, 418 assigned chloramphenicol and 426 gatifloxacin (figure 1). The baseline characteristics of the patients were similar in the two treatment groups (table 1). The proportion of patients with treatment failure was similar in the two treatment groups in patients with culture-positive disease (table 2). Of the five patients with persistent fever on day 10 in the gatifloxacin group (table 2), two became afebrile on day 11 and did not require rescue treatment. The other three patients were effectively treated with intravenous ceftriaxone 50 mg/kg per day in a single dose for 7 days. The five patients in the chloramphenicol group who needed rescue treatment were successfully treated with ofloxacin 20 mg/kg per day in two divided doses per day for 7 days. In all cases, rescue treatment was initiated on either day 10 or day 11.

**Table 1:** Baseline characteristics of patients according to treatment group

	**Chloramphenicol (n=418)**	**Gatifloxacin (n=426)**
Median (IQR) age (years)	15 (8–22)	16 (9–22)
Male sex	261 (62·4%)	279 (65·5%)
Median (IQR) weight (kg)	42 (20–51)	44 (23–53)
Median (IQR) duration of illness before admission (days)	5 (4–7)	5 (4–7)
Median (IQR) temperature at admission (°C)	38·95 (38·2–39·5)	38·90 (38·1–39·4)
Headache	375 (89·7%)	374 (87·8%)
Anorexia	323 (77·3%)	308 (72·5%)
Abdominal pain	181 (43·5%)	157 (37·1%)
Cough	145 (34·8%)	129 (30·4%)
Nausea	120 (28·7%)	136 (32·1%)
Vomiting	86 (20·7%)	81 (19·6%)
Diarrhoea	78 (18·8%)	79 (18·6%)
Constipation	60 (14·4%)	42 (9·9%)
Hepatomegaly	47 (11·2%)	66 (15·5%)
Splenomegaly	64 (15·3%)	55 (12·9%)
Median (IQR) haematocrit (%)	39 (36·0–43·5)	40 (36·0–43·0)
Median (IQR) leucocyte count (×10^9^/L)	6·4 (5·0–8·1)	6·2 (5·1–8·1)
Median (IQR) platelet count (×10^9^/L)	190 (162–219)	193 (165–232)
Median (IQR) AST (U/L)	46 (34–62)	44 (33–60)
Median (IQR) ALT (U/L)	29 (20–43)	30 (20–42)
*Salmonella typhi* isolated	125	124
*Salmonella paratyphi* A isolated	50	53
Positive pretreatment faecal cultures	20 (5·3%)	19 (5·1%)

AST=serum aspartate aminotransferase (normal range 12–30 U/L). ALT=serum alanine aminotransferase (normal range 13–40 U/L).

**Table 2:** Summary of primary and secondary outcomes for culture-positive patients (per-protocol analysis)

		**Chloramphenicol (n=175)**	**Gatifloxacin (n=177)**	**Comparison**
Total number of treatment failures*		14	12	HR 0·86 (95% CI 0·40 to 1·86), p=0·70
	Persistent fever at day 10	5	5	..
	Need for rescue treatment	5	3	..
	Microbiological failures	0	2	..
	Relapse until day 31	7	4	..
	Enteric fever related complications	0	0	..
Probability of treatment failure†		0·08 (95% CI 0·04 to 0·13)	0·07 (95% CI 0·03 to 0·11)	RD −0·01 (95% CI −0·07 to 0·04), p=0·64
Median time to fever clearance (days)†		3·95 (95% CI 3·68 to 4·68)	3·9 (95% CI 3·58 to 4·27)	HR 1·06 (95% CI 0·86–1·32), p=0·59
Microbiological failures‡		0/170 (0%)	2/167 (1%)	§p=0·24
Relapses until day 31		7	4	HR 0·56 (95% CI 0·16–1·91), p=0·35
	Number of culture confirmed relapses	5	3	..
	Number of syndromic relapses	2	1	..
Probability of relapse until day 31†		0·04 (95% CI 0·01 to 0·07)	0·02 (95% CI 0·00 to 0·05)	..
Relapses until day 62		10	9	HR 0·87 (95% CI 0·35 to 2·15), p=0·77
	Number of culture confirmed relapses	8	5	..
	Number of syndromic relapses	2	4	..
Probability of relapse until day 62†		0·06 (95% CI 0·02 to 0·10)	0·06 (95% CI 0·02 to 0·09)	..
Relapses after day 62 (all of which were syndromic)		4	10	..

HR=hazard ratio (based on Cox regression). RD=absolute risk difference (based on Kaplan-Meier estimates).

* Patients can have more than one type of treatment failure.

† Kaplan-Meier estimates.

‡ Only patients with a blood culture taken on day 8.

§ Based on Fisher's exact test.

Two patients with microbiological failure in the gatifloxacin group also had persistent fever, and responded well to ceftriaxone 50 mg/kg per day in a single daily dose for 7 days. All relapse patients, consisting of seven (five of whom were culture confirmed) in the chloramphenicol group and four (three of whom were culture confirmed) in the gatifloxacin group, were also treated with ofloxacin 20 mg/kg per day, and recovered.

**Figure 2:**
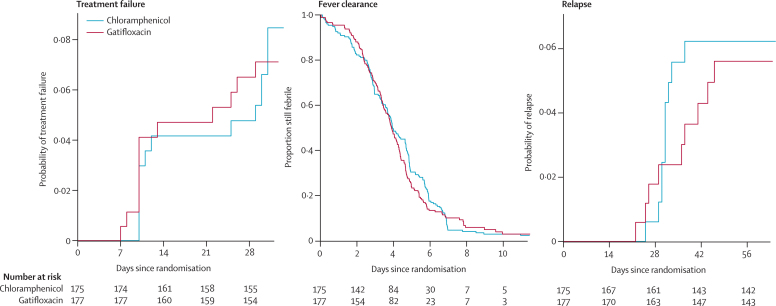
Kaplan-Meier estimates for time to treatment failure, fever clearance, and relapse for culture-positive patients

The secondary outcome measures, which included fever clearance time (median 3·95 days in the chloramphenicol group and 3·90 in the gatifloxacin group) and time to relapse until day 31 or day 62 also showed no significant difference between the groups (table 2). Only syndromic relapses were documented between day 62 and 6 months. Figure 2 shows the Kaplan-Meier estimates for the time to treatment failure, fever clearance, and relapse.

Stool samples at baseline were positive for *S typhi* or *S paratyphi* A in 16 (10%) of 157 patients in the chloramphenicol group and 14 (9%) of 160 patients in the gatifloxacin group. The proportion of positive stool samples at 1–6 months of follow-up was low in both groups: at 1 month, only three (2%) of 148 and none of 154 patients were stool-culture-positive in the chloramphenicol and gatifloxacin groups (p=0·12), respectively. At the end of 3 months, only one patient (in the chloramphenicol group) had a positive stool culture, and at 6 months no patients had a positive stool culture.

Table 3 shows the primary and secondary endpoints in all randomised patients, with the exception of patients who were mistakenly randomly allocated treatment or withdrew before the first dose of study treatment. There was a slightly greater risk of treatment failure in patients receiving chloramphenicol (p=0·09). Results in selected subgroups (table 4) suggest that this is primarily due to a higher failure rate of chloramphenicol in the culture-negative population, especially a higher rate of relapses until day 31 (nine [three confirmed, six syndromic] *vs* two [both syndromic]; HR of time to relapse=0·22, 95% CI 0·05–1·01, p=0·05). The median duration of chloramphenicol treatment was 9 days (IQR 8–11) in the culture-negative population, but there was not a significant association between the duration of treatment and the time to relapse (HR=0·93, 95% CI 0·66–1·30, p=0·66).

**Table 3:** Summary of primary and secondary outcomes for all patients

		**Chloramphenicol (n=418)**	**Gatifloxacin (n=426)**	**Comparison**
Total number of treatment failures*		26	15	HR 0·57 (95% CI 0·30–1·08), p=0·09
	Persistent fever at day 10	7	6	..
	Need for rescue treatment	6	4	..
	Microbiological failures	0	2	..
	Relapse until day 31	16	6	..
	Enteric fever related complications	0	0	..
Probability of treatment failure†		0·07 (95% CI 0·04 to 0·09)	0·04 (95% CI 0·02 to 0·06)	RD −0·03 (95% CI −0·06 to 0·00); p=0·07
Median time to fever clearance (days)†		2·69 (95% CI 2·44 to 2·85)	2·69 (95% CI 2·41 to 2·88)	HR 0·99 (95% CI 0·87 to 1·14); p=0·93
Microbiological failures‡		0/185 (0%)	2/181 (1%)	p§=0·24
Relapses until day 31		16	6	HR 0·37 (95% CI 0·14 to 0·94); p=0·04
	Number of culture confirmed relapses	8	3	..
	Number of syndromic relapses	8	3	..
Probability of relapse until day 31†		0·04 (95% CI 0·02 to 0·06)	0·02 (95% CI 0·00 to 0·03)	..
Relapses until day 62		23	12	HR 0·50 (95% CI 0·25 to 1·02); p=0·06
	Number of culture confirmed relapses	12	5	..
	Number of syndromic relapses	11	7	..
Probability of relapse until day 62†		0·06 (95% CI 0·04 to 0·08)	0·03 (95% CI 0·01 to 0·05)	..

HR=hazard ratio (based on Cox regression). RD=absolute risk difference (based on Kaplan-Meier estimates).

* Patients can have more than one type of treatment failure.

† Kaplan-Meier estimates.

‡ Only patients with a blood culture taken on day 8.

§ Based on Fisher's exact test.

**Table 4:** Comparison of treatment failure in the culture-positive and culture-negative population and selected subgroups

	**Chloramphenicol**	**Gatifloxacin**	**HR (95%CI)**	p for heterogeneity*
**Population**				
Culture positives	14/175	12/177	0·86 (0·40–1·86, p=0·70)	0·08
Culture negatives	12/243	3/249	0·25 (0·07–0·87, p=0·03)	..
**Pathogen**				
*Salmonella typhi*	11/125	8/124	0·73 (0·29–1·82, p=0·50)	0·51
*Salmonella paratyphi* A	3/50	4/53	1·32 (0·30–5·91, p=0·72)	..
**Age**				
Less than 16 years	18/222	10/217	0·58 (0·27–1·25, p=0·17)	0·98
16 years or older	8/196	5/209	0·59 (0·19–1·8, p=0·35)	..

* Heterogeneity was tested with a Cox regression model that included an interaction between treatment and subgroup.

There was no indication of treatment effect heterogeneity in the subgroups defined by pathogen or age (table 4).

Most adverse events were mild (grade 1 and 2; table 5). Adverse events were slightly more common in the culture-positive patients than the culture-negative patients. In the chloramphenicol group, 44 (25%) of 175 culture-positive patients experienced at least one adverse event (81 events in total). In the gatifloxacin group, 30 (16·9%) of 177 culture-positive patients experienced at least one adverse event (38 events in total). Three patients in the chloramphenicol group had a white-blood-cell count between 1500 and 1999×10^6^ cells per L on day 5–8, and had their chloramphenicol stopped. No grade 3 or 4 leucopenia was recorded (table 6). No grade 4 hypoglycaemias were recorded (table 7), and there were no life-threatening complications of enteric fever in this cohort.

**Table 5:** Adverse events: comparison of overall frequency and frequency of selected adverse events between the two treatment groups

	**Chloramphenicol (n=418)**		**Gatifloxacin (n=426)**		p value*
	Number of patients with event (%)	Number of events	Number of patients with event (%)	Number of events	
Any adverse event	99 (24%)	168	59 (14%)	73	0·0003
Abdominal pain	11 (3%)	12	8 (2%)	8	0·5
Acne	2 (<1%)	2	0	0	0·2
Anorexia	9 (2%)	10	1 (<1%)	1	0·01
Diarrhoea	24 (6%)	26	5 (1%)	5	0·0002
Dizziness	11 (3%)	11	2 (<1%)	2	0·01
Nausea	26 (6%)	29	9 (2%)	9	0·003
Oral candidiasis	4 (1%)	4	0	0	0·06
Vomiting	36 (9%)	39	35 (8%)	35	0·9
Weakness	4 (1%)	4	0 (0%)	0	0·06

All adverse events in this list were non-severe (ie, grade 1 or grade 2) except for one grade 3 dehydration in the chloramphenicol group and one grade 3 abdominal pain in the gatifloxacin group.

* Based on Fisher's exact test.

**Table 6:** Adverse events: leucopenia

	**Chloramphenicol (n=418)**	Gatifloxacin*(n=426)
**At baseline**		
Grade 1	2/411 (0·5%)	1/414 (0·2%)
Grade 2	0/411 (0%)	2/414 (0·5%)
**On day 8**		
Grade 1	4/403 (1·0%)	1/188 (0·5%)
Grade 2	3/403 (0·7%)	1/188 (0·5%)
**On day 15**		
Grade 1	1/351 (0·3%)	1/166 (0·6%)
Grade 2	0/351 (0%)	0/166 (0%)

Data are n (%) of patients tested. Grade 1 white blood cell (WBC) count 2000–2500×10^6^/L. Grade 2 WBC count 1500–1999×10^6^/L. No grade 3 or 4 leucopenia was recorded.

* Not all patients who received gatifloxacin had haematological tests on day 8 and day 15.

**Table 7:** Adverse events: dysglycaemia

	**Chloramphenicol (n=418)**	**Gatifloxacin (n=426)**	p value*
**Hyperglycaemia, grade 2**†			
At baseline	1/414 (0·2%)	2/422 (0·5%)	1·00
On day 2 to day 7‡	25/407 (6·1%)	42/414 (10·1%)	0·04
On day 8	0/402 (0%)	1/400 (0·3%)	0·50
On day 15	1/366 (0·3%)	0/351 (0·%)	1·00
On month 1	1/375 (0·3%)	0/383 (0·0%)	0·50
**Hypoglycaemia, grade 2 or worse**§			
At baseline	4/414 (1.0%)	4/422 (1·0%)	1·00
On day 2 to day 7‡	1/407 (0·3%)	1/414 (0·2%)	1·00
On day 8	2/402 (0·5%)	2/400 (0·5%)	1·00
On day 15	4/366 (1·1%)	3/351 (0·9%)	1·00
On month 1	3/375 (0·8%)	4/383 (1·0%)	1·00
HbA_1c_>6%			
On month 3	22/351 (6·3%)	20/359 (5·6%)	0·8

Data are n (%) of patients tested for abnormal blood glucose.

* Based on Fisher's exact test.

† Grade 2 non-fasting plasma glucose 161–250 mg/dL. No grade 3 or 4 hyperglycaemias were recorded.

‡ On days 2 to 7, all patients were monitored with fingerstick glucose testing.

§ Grade 2 non-fasting plasma glucose 40–54 mg/dL. One grade 3 hypoglycaemia (30–39 mg/dL) was recorded at baseline, and two on day 15 (one in each group). No grade 4 hypoglycaemias were recorded.

Of all the strains of *S paratyphi* A and *S typhi* isolated, 251 (73%) of 345 were nalidixic acid resistant, and two (<1%) were multidrug resistant (table 8). Both MDR strains were *S typhi* isolated from patients in the gatifloxacin group. Two *S paratyphi* A isolates were resistant to chloramphenicol, one of which was isolated from a patient in the gatifloxacin group and one of which was isolated from a patient in the chloramphenicol group.

**Table 8:** Antimicrobial susceptibility results: minimum inhibitory concentrations (MICs)*and resistance profile of*Salmonella paratyphi***A and***S typhi***isolates**

			***Salmonella paratyphi* A (n=103)**	***Salmonella typhi* (n=249)**	**p value**
Chloramphenicol					
	MIC 50 (μg/mL)		8·00	4·00	..
	MIC 90 (μg/mL)		12·00	8·00	<0·0001
		Range	2·00–64·00	1·50 to >256·00	..
Amoxicillin					
	MIC 50 (μg/mL)		1·00	0·50	..
	MIC 90 (μg/mL)		2·00	1·00	<0·0001
		Range	0·50–3·00	0·04 to >256·00	..
Cotrimoxazole					
	MIC 50 (μg/mL)		0·12	0·03	..
	MIC 90 (μg/mL)		0·19	0·06	<0·0001
		Range	0·02–0·38	0·01 to >32·00	..
Tetracycline					
	MIC 50 (μg/mL)		1·50	1·00	..
	MIC 90 (μg/mL)		2·90	2·00	<0·0001
		Range	0·50–8·00	0·38 to >256·00	..
Ceftriaxone					
	MIC 50 (μg/mL)		0·19	0·12	..
	MIC 90 (μg/mL)		0·25	0·19	<0·0001
		Range	0·12–0·38	0·05–0·25	..
Azithromycin					
	MIC 50 (μg/mL)		12·00	6·00	..
	MIC 90 (μg/mL)		16·00	12·00	<0·0001
		Range	1·00–48·00	0·38–24·00	..
Nalidixic acid					
	MIC 50 (μg/mL)		>256·00	>256·00	..
	MIC 90 (μg/mL)		>256·00	>256·00	<0·0001
		Range	1·50 to >256·00	0·38 to >256·00	..
Ciprofloxacin					
	MIC 50 (μg/mL)		0·50	0·25	..
	MIC 90 (μg/mL)		0·75	0·38	<0·0001
		Range	0·02–1·50	0·00–1·00	..
Ofloxacin					
	MIC 50 (μg/mL)		1·50	0·38	..
	MIC 90 (μg/mL)		2·00	0·50	<0·0001
		Range	0·06–6·00	0·02–4·00	..
Gatifloxacin					
	MIC 50 (μg/mL)		0·50	0·12	..
	MIC 90 (μg/mL)		0·50	0·19	<0·0001
		Range	0·02–1·50	0·00–1·00	..
Multidrug-resistant isolates			0 (0%)	2 (0·82%)	1·00
Nalidixic-acid-resistant isolates			92 (90·2%)	159 (65·43%)	<0·0001

* 102 *S typhi* and 243 *S paratyphi* A were available for MIC testing. MIC50/90=concentration at which 50% and 90% of the organisms, respectively, are inhibited. Multidrug resistance is defined as resistance to chloramphenicol, ampicillin, and co-trimoxazole. Comparisons are based on Wilcoxon test for continuous data and Fisher's exact test for categorical data.

In culture-positive patients, nalidixic acid resistance was significantly associated with a slower rate of fever clearance (HR 0·57, 95% CI 0·40–0·81, p=0·002) for patients on gatifloxacin, but there was no significant difference in speed of fever clearance between patients with nalidixic-acid-resistant strains and those without in the chloramphenicol group (0·80, 0·56–1·14, p=0·21).

## Discussion

Both chloramphenicol, which is a readily available drug in many resource-poor settings, and gatifloxacin, which is a newer generation fluoroquinolone, had excellent efficacy in the treatment of culture-positive enteric fever, and both drugs had a favourable side-effect profile. Gatifloxacin did as well as, but was not superior to, chloramphenicol in an area with a high proportion (73%) of nalidixic-acid-resistant *S typhi* and *S paratyphi* A strains, but almost no chloramphenicol resistance.

With 844 patients analysed (figure 1), this is to our knowledge the largest randomised controlled trial in enteric fever, and the biggest trial comparing chloramphenicol with a fluoroquinolone. This is also the first trial to compare chloramphenicol to a fluoroquinolone in a predominantly paediatric population (table 1). We also assessed the—to our knowledge—largest population of blood-culture-negative patients with enteric fever. In patients who had blood-culture-negative syndromic enteric fever, both drugs were effective, but gatifloxacin was more effective in reducing syndromic clinical relapse.

There are underlying technical issues for typhoid and enteric fever treatment trials. One of the central limitations is the low sensitivity of the blood culture technique, which is estimated to be between 40% and 50%.^22^ That most patients with enteric fever are categorised as syndromic, and treated empirically without a definitive diagnosis for enteric fever, is therefore not surprising. For the same reason, syndromic relapse was included as an outcome event in the a-priori defined analysis plan in this study.

The antibiotics used in this trial show different pharmacological properties. Gatifloxacin has important features likely to help with treatment adherence compared with chloramphenicol: gatifloxacin only needs to be taken once a day for 7 days, whereas chloramphenicol requires four doses per day for 14 days. There was no difference between the two drugs in terms of treatment failure and fever clearance time in the culture-positive group; however, the adverse effects profile showed that anorexia, nausea, diarrhoea, and dizziness, were significantly worse in the chloramphenicol group (table 5).

We monitored blood glucose levels closely in both treatment groups chiefly because of a recent Canadian, retrospective case-control study of 1·4 million elderly individuals (mean age 77) that showed that gatifloxacin was associated with dysglycaemia.^23^ After this report, gatifloxacin was withdrawn from the US and Canadian markets. In our trial, between day 2 and day 7, the proportion of patients with a high (grade 2; 161–250 mg/dL) non-fasting blood glucose on finger-stick testing was higher in the gatifloxacin group versus the chloramphenicol group. However, there was no difference on days 15 and days 30. Similarly, at the end of 3 months, HbA_1c_ concentrations were not different in the two groups (table 7). Additionally, previous studies using gatifloxacin in a younger population have not reported clinically relevant dysglycaemia.^24^ Finally, in another study comparing gatifloxacin with ofloxacin for the treatment of enteric fever that we are doing (ISRCTN63006567), we have not recorded any dysglycaemia. The gatifloxacin-associated dysglycaemia in the Canadian study might be attributed to an age-related decrease in renal function in elderly patients receiving gatifloxacin, and there might well be a pharmacokinetic or pharmacodynamic rationale for a potential age-related dose reduction.^25^ Treatment options for enteric fever are clearly limited. Gatifloxacin is an efficacious drug for the treatment of enteric fever in young and otherwise healthy patients, and should be available for indication in this neglected disease. It would be prudent not to use gatifloxacin in patients over 50 years of age, or in patients with comorbidities such as diabetes or renal failure.

Most enteric fever trials are done in an inpatient setting, which does not reflect reality in developing countries, where most uncomplicated enteric fever treatment is done in an outpatient setting.^1^, ^8^ Our trial was completed in an outpatient setting with the help of CMAs, as described in our earlier trial.^16^ This model is more applicable to developing countries.

A very attractive feature, especially for resource-poor settings, is the inexpensiveness of the antibiotics studied here. The average price for a 14-day treatment course with chloramphenicol was US$7. The average price for a 7-day treatment with gatifloxacin was US$1·5.

A recent Cochrane review (panel) of fluoroquinolones for the treatment of enteric fever pointed out the weaknesses of typhoid fever treatment trials that have small sample sizes, inadequate randomisation and concealment, incomplete follow-up, and a lack of paediatric patients and standardised endpoints.^7^ We tried to address these criticisms by recruiting a large sample of patients, by percisely defining our endpoints, and by attempting to reduce bias within the limits of an open trial.

Panel **Research in context**

**Systematic review**

We searched Medline for the terms “gatifloxacin”, “chloramphenicol”, “clinical trial”, and “typhoid/enteric fever”. We also identified relevant articles from a recent Cochrane review,^7^ WHO typhoid guidelines,^22^ and a recent meta-analysis of fluoroquinolones versus other antibiotics in the treatment of typhoid fever.^26^ There were ten trials^27^, ^28^, ^29^, ^30^, ^31^, ^32^, ^33^, ^34^, ^35^, ^36^ in the meta-analysis that compared fluoroquinolones with chloramphenicol. Multidrug-resistant strains were absent in all but one trial,^27^ and nalidixic acid resistance was only reported in one trial^27^ in which there were no nalidixic-acid-resistant strains. The meta-analysis concluded that fluoroquinolones were not significantly different from chloramphenicol for clinical failure or microbiological failure in an adult population. However, the sample sizes of the trials included in the analysis were small, and there was a paucity of paediatric data. There were only two previous trials of gatifloxacin for the treatment of uncomplicated enteric fever: one from Nepal and one from Vietnam.^16^, ^17^

**Interpretation**

Gatifloxacin was not better than chloramphenicol in children and adults in Nepal with enteric fever. Both gatifloxacin and chloramphenicol showed similar efficacy in the treatment of blood-culture-positive enteric fever in a setting with strains of *S typhi* and *S paratyphi* A fully sensitive to chloramphenicol and resistant to nalidixic acid. Our trial showed that both in the adult and paediatric population gatifloxacin was not better than chloramphenicol. However, in a developing-country setting like Nepal in a young population where this disease predominates, gatifloxacin should be the preferred choice because of its shorter treatment duration, fewer adverse events, and lower cost in the treatment of enteric fever.

Two other trials used gatifloxacin for the treatment of enteric fever (panel). 16, 17 The first trial compared gatifloxacin to cefixime, and enrolled children and adult outpatients in Nepal.^16^ This trial had to be prematurely stopped on the advice of the independent data safety monitoring committee because of the poor performance of cefixime. There was a high rate of overall treatment failure (persistent fever at day 7, relapse and death) with 29 (38%) of 70 patients failing in the cefixime group compared with three (3%) of 88 patients in the gatifloxacin group (HR 0·08, 0·03–0·28, p<0·001). There was one death in the cefixime group.

The second trial compared gatifloxacin with azithromycin, and was done in paediatric and adult in-patients in Vietnam.^17^ There was no statistical difference between the two antibiotics, and both showed excellent efficacy. The median fever clearance times were 106 h in both groups. 13 (9%) of 145 patients in the gatifloxacin group had overall treatment failure as did 13 (9%) of 140 in the azithromycin group (HR 0·93, 0·43–2·0, p=0·85). Both trials were done in regions with high rates of nalidixic-acid-resistant strains: 83% in Nepal and 96% in Vietnam. In previous trials in Vietnam, patients treated with the older generation fluoroquinolone ofloxacin given at 20 mg/kg per day showed high clinical failure rates of 36% (23 of 63 patients) and prolonged mean fever clearance times of 8·2 days (95% CI 7·2–9·2 days).^37^

Gatifloxacin is not superior to chloramphenicol in terms of efficacy. However, on the basis of its shorter treatment duration, fewer adverse events, and lower cost, gatifloxacin should be the preferred treatment of enteric fever in developing countries.
